# Pan-Vertebrate Toll-Like Receptors During Evolution

**DOI:** 10.2174/138920208786241234

**Published:** 2008-11

**Authors:** Hiroyuki Oshiumi, Aya Matsuo, Misako Matsumoto, Tsukasa Seya

**Affiliations:** Department of Microbiology and Immunology, Hokkaido University Graduate School of Medicine, Sapporo 060-8638, Japan

## Abstract

Human toll-like receptors (TLRs) recognize pathogen-associated molecular patterns (PAMPs) to raise innate immune responses. The human TLR family was discovered because of its sequence similarity to fruit fly (*Drosophila*) Toll, which is involved in an anti-fungal response. In this review, we focus on the origin of the vertebrate TLR family highlighted through functional and phylogenetic analyses of TLRs in non-mammalian vertebrates. Recent extensive genome projects revealed that teleosts contain almost all subsets of TLRs that correspond to human TLRs (TLR1, 2, 3, 4, 5, 7, 8, and 9), whereas the urochordate *Ciona* *intestinalis* contains only a few TLR genes. Therefore, mammals likely obtained almost all TLR family members at the beginning of vertebrate evolution. This premise is further supported by several functional analyses of non-mammalian TLRs. We have summarized several teleost TLRs with unique properties distinct from mammalian TLRs to outline their specific roles. According to *Takifugu rubripes* genome project, the puffer fish possesses fish-specific TLR21 and 22. Surprisingly, phylogenetic analyses indicate that TLR21 and 22 emerged during an early period of vertebrate evolution in parallel with other TLRs and that the mammalian ancestor lost TLR21 and 22 during evolution. Our laboratory recently revealed that TLR22 recognizes double-strand RNA and induces interferon production through the TICAM-1 adaptor, as in TLR3, but unlike TLR3, TLR22 localizes to the cell surface. Therefore, differential expression of TLR3 and TLR22, rather than simple redundancy of RNA sensors, may explain the effective protection of fish from RNA virus infection in the water. In this review, we summarize the similarities and differences of the TLR family in various vertebrates and introduce these unique TLRs for a possible application to the field of clinical practices for cancer or virus infection.

## MAMMALIAN TLR FAMILY 

Mammalian toll-like receptors (TLRs) were discovered because of their sequence similarities to fruit fly (*Drosophila*) Toll, which plays a crucial role in both anti-fungal protection and dorsal and ventral pattern establishment in the embryo [1, 2]. The human genome encodes 10 TLRs, each of which recognizes different pathogen-associated molecular patterns (PAMPs); they are not involved in development. Among human TLRs, the function of TLR4 was the first to be revealed *via *the analysis of C3H/HeJ mice, which have a defective response to LPS endotoxin [3, 4]. Inquiry into the genetic basis of LPS resistance revealed a single locus (LPS) at which homozygosity for a codominant allele (LPSd) caused the endotoxin-unresponsive site. The codominant LPSd allele of C3H/HeJ mice corresponds to a missense mutation in the TLR4 ORF [5]. Now the functions of almost all human TLRs are known: TLR3, 5, 7, and 8 or 9 recognize viral double-strand RNA, flagellin protein, single-strand RNA, or CpG, respectively [5-11], and TLR2 and 6 and TLR2 and 1 form a heterodimer to recognize diacyl or triacyl lipopeptides, respectively [12-15]. Their functions were determined mainly by knockout mice analyses. Human patients who harbor mutations of the TLR gene exhibit abnormal innate immune responses. Missense mutations of TLR3 occur in patients with herpes simplex encephalitis, and the TLR3 allele confers dominant hyporesponsiveness to a TLR3 ligand polyI:C in fibroblasts [16]. IRAK4 is a component of TLR signaling, and mutation of IRAK-4 is found in children with recurrent infections with a poor inflammation response whose blood and fibroblasts do not respond to TLR ligands [17]. 

The innate immune system is composed of Tolls in invertebrates and TLRs in vertebrates, and distinct differences exist between mammalian TLRs and the arthropod Toll family. For example, mammalian TLRs directly recognize PAMPs, whereas *Drosophila *Toll receives the PAMP signal indirectly through endogenous proteins. Recently, crystal structures of mammalian TLRs have been reported, which illustrate the mammalian process. Kim, Lee and their colleagues described the crystal structure of the TLR4 extracellular domain in complex with MD-2 bound to eritoran, an analog of LPS, that antagonizes TLR4 signaling [18]. Jin, Lee and their colleagues reported the crystal structure of the complex of TLR1 and 2 extracellular domains bound to a synthetic lipopeptide agonist Pam3CSK4 [19]. In contrast, the fruit fly utilizes another strategy to recognize PAMPs. For example, the peptidoglycan-recognition protein PGRP-SA is a soluble-receptor that directly recognizes Gram-positive bacteria [20]. Microbial recognition by these receptors triggers the zymogen cascades that lead to the cleavage of the proform Spatzle into an activated form and ultimately to amplifying the Toll responses. The active Spatzle binds Toll and activates a signaling pathway that produces anti-bacterial peptides [21]. Phylogenetic analyses show that no orthologous relationship exists between mammalian and fruit fly Toll family members, and the two families supposedly developed independently during evolution [22, 23]. Thus, the next question is when the current human TLR subsets appeared during evolution.

## VERTEBRATE TLR FAMILY

The draft genome sequence of the puffer fish (*Takifugu* *rubripes*) is firstly reported to be non-mammalian vertebrate genome [24], and its information provided a complete view of the puffer fish TLR family of genes. Interestingly, the puffer fish genome encodes orthologues of human TLR1, 2, 3, 5, 7, 8, and 9. TLR4 does not exist in this genome; however, a TLR4-like gene was reported in the genomes of several other teleosts, such as *Danio* *rerio* [22], although the functional features of this gene remain undetermined. Thus, the common ancestor of humans and teleosts is predicted to have lived in the Devonian Period and to have had TLR1, 2, 3, 4, 5, 7, 8, and 9 genes. In contrast to the teleost genome, the genome of the urochordate *Ciona* *intestinalis* contains only a few TLR genes [25], and that of *S*. *purpuratus*, a sea urchin, possesses ~200 TLRs with uncharacterized functions that are incomparable with the human TLR subsets [26, 27]. Interestingly, the lamprey (*Lamprey* *japonica*, a jawless fish) possesses TLR14a and 14b genes [28]. TLR14 is a member of the TLR2 subfamily, and the gene is present in the genome of teleosts and amphibians [22, 28]. This suggests that the current TLR subsets emerged before the mammalian ancestor diverged from the jawless fish ancestor. Based on chordate genome information and phylogenetic analyses, the prototype of the current human TLR family likely emerged during the Cambrian period when the vertebrate ancestor emerged [22].

Generally speaking, proteins from orthologous genes do not necessarily share common functions. Do the TLR orthologs conserved in fish and mammals have the same functions? Recent functional analyses revealed that the vertebrate orthologous gene products have the same functions as their human counterparts. For example, the rainbow trout TLR5 gene is up-regulated by stimulation with recombinant flagellin proteins, as shown in teleost IL-1 βR. Interestingly, rainbow trout encodes another TLR5 gene, which lacks the transmembrane region and whose products are liberated from cells. The soluble TLR5s promote a teleost membrane type TLR5 chimera protein that mediates NF- κ B activation in human cells. Therefore, teleost TLR5 is a receptor for flagellin, as is the case for human TLR5 [29]. Another teleost TLR, *Takifugu* *rubripes* TLR3, responds to the same ligand as does human TLR3. Recently, our laboratory showed that the *Takifugu* *rubripes* TLR3 gene is up-regulated by polyI:C stimulation in teleost cell lines, as is true for the human TLR3 gene, whose expression is induced in several cell species and cell lines [30]. TLR3 expression in both human and fish cells provides a responsiveness to the polyI:C and double-strand RNA [30]. Like mammalian TLR3, the teleost TLR3 is localized in the intracellular compartments and is largely merged with an ER marker, Calnexin, in HeLa cells [30]. These data indicate that both human and teleost TLR3 encompass a common RNA-sensing role against virus infection in the innate immune system. Therefore, the common ancestor of humans and teleosts likely contained the double-strand RNA recognition system involving TLR3. These functional conservations are also observed in other non-mammalian vertebrates. 

Like human TLR2, chicken (*Gallus* *gallus*) TLR2 responds to lipoproteins [31]. The chicken likely has the following additional TLR2 subfamily members: TLR2-type1 and 2, TLR1-type1 and 2. Like human TLR2 subfamily members of TLR1, 2, and 6, the avian TLR2 subfamily members form heterodimers and TLR assembly is required for the recognition of PAMPs [32]. These functional similarities support the notion that TLR functions are conserved in vertebrates and that the vertebrate common ancestor had established the innate immune system that detects lipoprotein, peptidoglycan, LPS, flagellin, double- or single strand RNA, and CpG DNA as PAMPs before the vertebrate species diverged. 

## NON-PRIMATE TLR FAMILY

Interesting differences among vertebrate TLR families also exist. We previously discussed the presence of non-mammalian TLRs (TLR21 and 22) in the puffer fish genome [23], and they are expressed in various tissues, suggesting that they are not pseudogenes but rather functional in the puffer fish [23]. Subsequent analyses revealed presence of other non-mammalian TLRs, such as TLR23 and TLR14 and the non-primate TLRs TLR11, 12, and 13 [22]. Phylogenetic tree analyses showed that some of those TLRs are derived from the TLR2 lineage. The human TLR2 subfamily includes TLR1, 6, and 10, and the TLR2 subfamily is known to be divergent in several avian and teleost species. For instance, the chicken has two TLR2 and two TLR1 genes. The teleost genome contains several non-mammalian TLR2 subfamily members, such as TLR14, which is also found in the lamprey, as described above. Unlike those TLR2 subfamily members, TLR21 and 22 are not included in any clade of human TLRs, and they likely originated around the Cambrian period; this indicates that the human ancestor possessed TLR21 and 22 genes [22, 23]. 

What are the roles of non-mammalian or non-primate TLRs? Cells expressing TLR11, a non-primate TLR, fail to respond to any primate TLR ligands, but they do respond to uropathogenic bacteria [33]. Analysis of TLR11 knockout mice revealed the importance of TLR11 in IL-12 production from dendritic cells [34]. Recently, we discovered the function of a non-mammalian TLR, TLR22: *Takifugu* *rubripes* TLR22 expression confers responsiveness to double-strand RNA or polyI:C on transfected cells as human TLR3 responds [30]. This is surprising because teleosts also have TLR3, and indeed the teleost TLR3 protein responds to double-strand RNA in a manner similar to that of teleost TLR22. The question is why teleosts have two double-strand RNA recognition receptors.

We revealed two functional differences between TLR3 and TLR22. The first is that TLR3 and TLR22 discriminate between size-differences of double-strand RNA. TLR3 prefers to recognize short dsRNA (< 1 kbp), whereas TLR22 prefers long dsRNA (< 1 kbp) [30]. TLR3 and TLR22 also differ in their localization profiles. TLR22 is located on the cell surface membrane [30], whereas the four human TLRs (TLR3, 7, 8, and 9) that recognize nucleic acids are localized in the early endosome or ER in myeloid cells [35-37]. Therefore, TLR22 is the only TLR that can recognize nucleic acids on the cell surface and transmit signals to induce cytokines.

The importance of TLR22 in teleosts is shown by its activity against RNA viruses. Several pathogenic RNA viruses infect teleosts. One of the birnaviruses, IPNV, causes necrosis in the pancreas of teleosts, and its genome is double-strand RNA [38]. When RTG-2 cells (derived from the kidney of rainbow trout) expressing TLR22 are infected with IPNV, TLR22 expression confers resistance to the virus to the RTG-2 cells.

**Fig. (1). Two double-strand RNA recognition pathways in teleosts:**
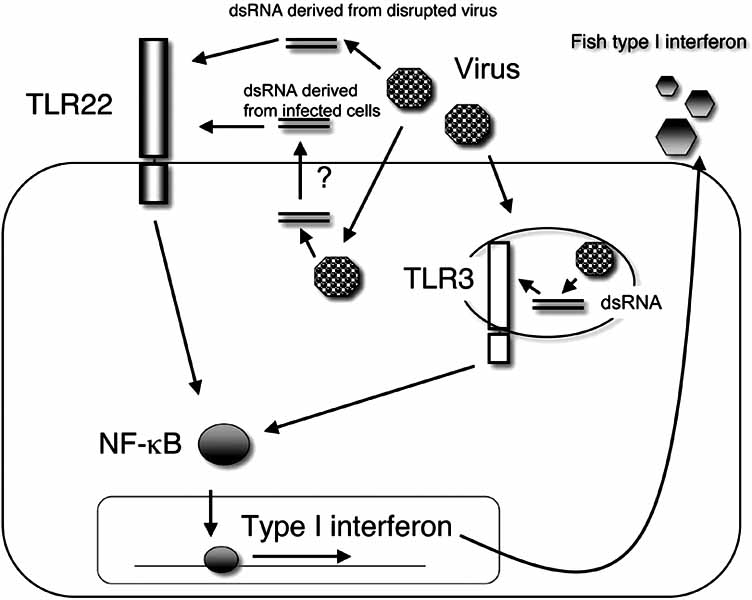
TLR22 localizes at the cell surface and recognizes double-strand RNA derived from viruses. Teleost TLR3 proteins reside in the intracellular compartment and are expected to localize at the early endosome, as in human TLR3. When virions are disrupted and their genome RNA flows out to the extracellular space, one might surmise that the viral double-strand RNA is recognized by TLR22. In another case, when the viral RNA in the cytoplasmic region is exported from the cytoplasm to the intercellular space by exocytosis, the double-strand RNA can be recognized by TLR22. Teloeost TLR3 recognizes viral RNA at the early endosome as in human TLR3. Both TLRs can transmit the signal to induce type I interferon, which exerts anti-virus properties.

TLR22 is widely conserved among teleosts and amphibians, but extensive genome projects failed to reveal the presence of the TLR22 gene in avian or mammalian genomes. Therefore, it seems likely that TLR22 is required for vertebrates that live in the water [30]. TLR22 is ubiquitously expressed in puffer fish tissues [23], but tissue-specific expression, with strong expression in the head and kidney, mild expression in the trunk, spleen, and gill, and undetectable expression in the intestine, liver, brain, and skin, has been reported for the Japanese founder (*Paralichthys olivaceus*) [39]. This finding illustrates that the expression pattern is different among different teleosts. Interestingly, both the puffer fish and Japanese flounder TLR22 genes are up-regulated by stimulation with polyIC, which is a synthetic analog of double-strand RNA [39]. Therefore, the TLR22 function seems to be conserved among teleosts. Teleosts possess two viral RNA-recognizing TLRs: TLR3 and TLR22. Double-strand RNA derived from RNA virus is recognized by TLR22 on the cell surface and simultaneously by TLR3 in the early endosome (Fig. **1**).

In addition to TLR22, vertebrates have other non-mammalian TLRs, such as TLR21 [22, 23]. TLR21 is present not only in teleosts but also in *Xenopus* *tropicalis* and the chicken [22]. Phylogenetic analysis indicates that TLR21 is a member of the TLR11 subfamily, which includes mouse TLR12 and 13 and teleost TLR20 [22, 40]. Like TLR22, TLR21 is also widely expressed in various tissues, such as the liver, spleen, kidney, skin, and gill in both the puffer fish and catfish (*Ictalurus punctatus*) [23, 40]. The function of TLR21 remains to be determined, but we expect that TLR21 responds to the PAMPs that are not recognized by any other TLRs so far described. Considering that the puffer fish does not possess the TLR4 gene, teleosts should have another mode of detecting PAMPs derived from the Gram-negative bacteria because there are many pathogenic Gram-negative bacteria that infect teleosts in the water. Thus, the agonist candidate for TLR21 may be a component of the Gram-negative bacteria that induces a response through this non-mammalian TLR.

## EVOLUTION OF THE VERTEBRATE TLR FAMILY 

**Fig. (2). Evolution of the vertebrate TLR family:**
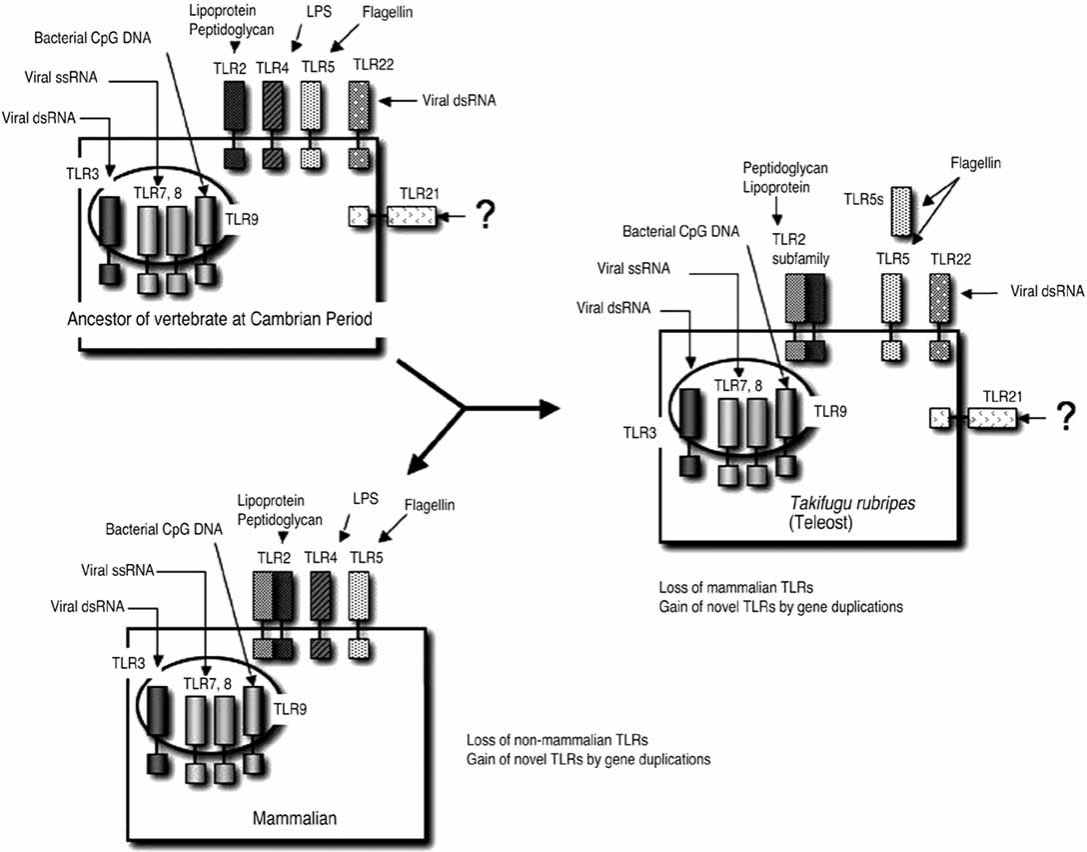
We expect that in the Cambrian period, the vertebrate ancestor possessed at least nine TLR family members: TLR2, 3, 4, 5, 7, 8, 9, 21, and 22. Those TLRs would have responded to PAMPs such as dsRNA, ssRNA, CpG DNA lipoprotein, peptidoglycan, LPS, flagellin, or other unknown PAMPs. Current TLR members in fish and mammals are expected to have been derived from the TLR family members in the Cambrian common ancestor. During evolution, mammalian ancestors obtained novel TLR members by gene duplication, especially in the TLR2 subfamily. On the other hand, both lineages have lost several members for unknown reasons.

The phylogenetic tree of vertebrate TLR family members strongly supports the notion that the non-mammalian vertebrate TLRs emerged during the Cambrian period together with other mammalian TLRs, and thus the human ancestor should have possessed both current TLR subsets and those of non-mammalian vertebrates. Based on our knowledge of the functional coverage of the vertebrate TLR family members, the expected TLR subsets that the vertebrate common ancestor possessed would include at least the following 10 TLR members: TLR2, 3, 4, 5, 7, 8, 9, 11, 21, and 22. Before the evolution of mammals, gene duplications would have occurred, especially in TLR2 subfamily members. Furthermore, some TLR genes were lost in some lineages, although the reason is as yet unknown. For example, TLR21 was diminished in the mammalian lineage, and TLR22 was lost when the mammalian ancestor began to live on land (Fig. **2**). Why did the human ancestor lose TLR21 and 22 during evolution? We suggest two possible answers to this question. First, mammals obtained another way to detect PAMPs so that non-mammalian TLRs became dispensable in the innate system. This scenario is conceivable because the mammalian acquired system is far more sophisticated than that of teleosts. Second, the mammalian lineage happened to lose the non-mammalian TLRs. This is not surprising because losses of genes, which are useful for their descendant, sometimes occurred during vertebrate evolution. For example, the vertebrate ancestor likely possessed six types of opsin gene for light sensing, but the mammalian ancestor lost three of the pigment genes since their divergence from reptiles [41]; thus, many mammalian species are less sensitive to the difference of light wavelength compared to other non-mammalian vertebrates. If mammals had successfully reproduced TLR22 again in their genomes, human innate immunity would have become better than the current system. Whether this is the case or not seems to reflect the reason why human ancestors lost the genes. If the first hypothesis is true, the addition of TLR22 would not affect mammalian innate immune system. On the other hand, according to the second hypothesis, it is predicted that the addition of TLR22 leads to addition of another mechanism of sensing viral infection on the cell surface to the mammalian innate immune system, and therefore the addition of TLR22 will provide a resistance to some kind of virus infection. Our laboratory currently is conducting research to find a reply to this question: We are trying to produce TLR22 transgenic mice, which will tell us why human lost TLR22 during evolution.
